# *Period2* gene mutant mice show compromised insulin-mediated endothelial nitric oxide release and altered glucose homeostasis

**DOI:** 10.3389/fphys.2012.00337

**Published:** 2012-08-23

**Authors:** João M. Carvas, Ana Vukolic, Gautham Yepuri, Yuyan Xiong, Katja Popp, Isabelle Schmutz, Sylvie Chappuis, Urs Albrecht, Xiu-Fen Ming, Jean-Pierre Montani, Zhihong Yang

**Affiliations:** ^1^Faculty of Science, Division of Physiology, Department of Medicine, University of FribourgFribourg, Switzerland; ^2^Faculty of Science, Department of Biology, Biochemistry, University of FribourgFribourg, Switzerland

**Keywords:** blood vessel, circadian gene, glucose, glycogen, insulin, nitric oxide, obesity, signaling

## Abstract

Period2 (Per2) is an important component of the circadian clock. Mutation of this gene is associated with vascular endothelial dysfunction and altered glucose metabolism. The aim of this study is to further characterize whole body glucose homeostasis and endothelial nitric oxide (NO) production in response to insulin in the *mPer2*^*Brdm1*^ mice. We show that *mPer2*^*Brdm1*^ mice exhibit compromised insulin receptor activation and Akt signaling in various tissues including liver, fat, heart, and aortas with a tissue-specific heterogeneous diurnal pattern, and decreased insulin-stimulated NO release in the aortas in both active and inactive phases of the animals. As compared to wild type (WT) mice, the *mPer2*^*Brdm1*^ mice reveal hyperinsulinemia, hypoglycemia with lower fasting hepatic glycogen content and glycogen synthase level, no difference in glucose tolerance and insulin tolerance. The *mPer2*^*Brdm1*^ mice do not show increased predisposition to obesity either on normal chow or high fat diet compared to WT controls. Thus, mice with *Per2* gene mutation show altered glucose homeostasis and compromised insulin-stimulated NO release, independently of obesity.

## Introduction

The master circadian clock residing in the suprachiasmatic nucleus (SCN) of the hypothalamus and the clocks in peripheral tissues regulate many physiological functions including glucose metabolism and cardiovascular functions (Albrecht, 2004). The molecular clock contains a set of core genes—*Clock, Bmal1, Cry1-2, Per1-3*, and nuclear receptors (*Rev-erb*α, *ROR*α) (Albrecht, 2004). Through positive and negative transcriptional and post-translational feedback loops, they generate an endogenous rhythm of intracellular protein expression, leading to rhythmic cell and tissue function that oscillates over approximately 24 h (Albrecht, 2004).

Several lines of evidence suggest that circadian misalignment and circadian clock gene disruption in humans and animals impact the comorbidities of metabolic disorders and cardiovascular risks such as obesity, diabetes, and atherosclerosis (Scheer et al., 2009; Paschos and Fitzgerald, 2010; Huang et al., 2011). These disease states are associated with chronic low grade inflammation manifested by tissue macrophage infiltration and local production of pro-inflammatory cytokines (Weisberg et al., 2003; Xu et al., 2003; Lumeng and Saltiel, 2011). Mice with a mutation in the *Clock* or *BMAL1* gene show phenotypes resembling the metabolic syndrome, including predisposition to obesity, hyperglycaemia, and hyperlipidaemia (Rudic et al., 2004; Shimba et al., 2005; Turek et al., 2005; Lamia et al., 2008). The effects of a mutation in the *Per2* gene on obesity and metabolic profiles are, however, inconsistent and contradictory. While one study reports that mice with a *Per2* gene disruption are prone to obesity (Yang et al., 2009; Grimaldi et al., 2010), a recent study using *mPer2*^*Brdm1*^/Rev-Erbα^−/−^ double mutant mice suggests that the *Per2* gene is involved in hepatic glucose metabolism engaging a mechanism involving nuclear receptors (Schmutz et al., 2010). However, whether mice with a *Per2* gene mutation alone exhibit altered whole body glucose homeostasis and insulin responses has not been reported.

Besides regulating glucose homeostasis, insulin also exerts profound effects on vascular endothelial function by enhancing the release of the vasoprotective molecule nitric oxide (NO) through the protein kinase Akt, which phosphorylates and activates endothelial NO-synthase (eNOS) (Yu et al., 2011). Defects of insulin signaling therefore result in insulin resistance, alterations in glucose tolerance, and vascular endothelial dysfunction, which is often seen in the metabolic syndrome, a cluster of cardiovascular risk factors (Yu et al., 2011).

Given the contradictory results on predisposition of *Per2* mutant mice to obesity (Yang et al., 2009; Grimaldi et al., 2010), we further characterized in this study the whole body glucose metabolic and vascular phenotypes of the *Per2* mutant mice.

## Materials and methods

### Materials

All chemicals including those for immunoblotting were obtained from Sigma (Buchs, Switzerland), unless otherwise indicated. Anti-Akt (#9272) and phospho-Akt-Ser473 (#9271), and anti-glycogen synthase (#3886) were from Cell Signaling; anti-IRβ (sc-711) and phospho-IRβ-Tyr1162/1163 were from Santa Cruz Biotechnology, Inc., Alexa Fluor680-conjugated anti-mouse IgG (A21057) were from Molecular Probes/Invitrogen (Lucerne, Switzerland); IRDye800-conjugated anti-rabbit IgG (926-32211) were from LI-COR Biosciences (Bad Homburg, Germany); 4,5-diaminofluoresceine acetate (DAF-2DA) was from VWR international SA (Dietikon, Switzerland).

### Animals

The *mPer2*^*Brdm1*^ mutant mice were generated and propagated as previously described (Zheng et al., 1999). Briefly, mice were bred from pairs heterozygous for *Per*2^*Brdm*1^ in mixed background of C57BL/6, 129SvEvBrd strains. All animals were maintained in a temperature and humidity-controlled facility (temperature: 22 ± 1°C; humidity: 55 ± 5%) with a 12-h light–dark cycle (LD12:12 cycle) and fed *ad libitum* with normal chow (NC; energy content: 12% fat, 28% protein, and 60% carbohydrate; UAR, Epinay sur Orge, France). For the study, age matched (5–8 months) wild type (WT) littermates and *mPer2*^*Brdm1*^ male mice were used. Before experiments, food was removed for six hours. Mice were sacrificed either at Zeitgeber time (ZT9) (9 h after lights on) or at ZT15 (3 h after lights off). To study susceptibility of the animals to obesity, the WT and *mPer2*^*Brdm1*^ mutant mice were fed a high fat diet (HFD; composition: 55% fat, 21% protein, and 24% carbohydrate; Harlan Teklad TD 93075; Horst, Netherlands) starting at the age of 12 week old for 10 weeks. Body weight was measured weekly. Animal experimental protocols were approved by the Ethical Committee of Veterinary Office of Fribourg (A178/07), Switzerland. To measure blood glucose concentrations under fasting and refeeding conditions, the WT and *mPer2*^*Brdm1*^ mice were food deprived for 6 h followed by refeeding with normal chow for 90 min. Blood glucose levels were then measured with a One-Touch Glucose Monitoring System Ascensia Contour (Bayer) under the fasting and refeeding conditions, respectively. In another series of experiments, food intake was measured during 3 h of refeeding in the two groups of the mice.

### Insulin tolerance test (ITT) and glucose tolerance test (GTT)

Insulin tolerance test (ITT) and glucose tolerance test (GTT) were performed after 6 h of fasting at ZT9 or ZT15. 0.5 mU/g insulin (Actrapid HM, Novo-Nordisk) or 1 g/kg glucose (Masuzaki et al., 1999) was injected intraperitoneally to the age-matched WT and *mPer2*^*Brdm1*^ mice for ITT or GTT, respectively. Blood glucose levels were measured with a One-Touch Glucose Monitoring System Ascensia Contour (Bayer) at defined times. Moreover, fasting plasma insulin concentration was measured with ELISA by Services of the Metabolic Platform at Metabolic Evaluation Facility (MEF), Faculty of Biology and Medicine, University of Lausanne, Switzerland.

### Tissue insulin signaling

Mouse thoracic aortas were isolated from both wild-type and *mPer2*^*Brdm1*^ mutant mice following intraperitoneal injection of ketamin (1.0 mg/kg body weight, i.p.) plus xylazin (10 mg/kg body weight). Aortas were placed in cold Krebs-Ringer buffer with the following composition (mmol/L): 118 NaCl, 4.7 KCl, 2.5 CaCl_2_, 1.2 MgSO_4_, 1.2 KH_2_PO_4_, 25 NaHCO_3_, 0.026 EDTA, and 5.0 D-glucose. The aortas were cleaned of the perivascular adipose tissue and loose connective tissue and cut into ring segments. For analyzing insulin-stimulated signaling in the vasculature, the aortic segments were incubated in Krebs buffer at 37°C aerated with 95% O_2_ and 5% CO_2_. After 30 min of equilibration, insulin, or vehicle was added to the vascular rings to reach a final concentration of 100 nmol/L for 5 or 20 min (Yuen et al., 2009). Aortic tissues were immediately snap-frozen in liquid nitrogen and kept in −80°C till use. Signal transductions stimulated by insulin in the aortas were analyzed by immunoblotting.

To investigate the insulin signaling in other tissues, mice were starved for 6 h and recombinant insulin (0.5 mU/g of body weight) was then injected intraperitoneally. Mice were sacrificed 5 min post stimulation (Frangioudakis et al., 2005). Tissues, i.e., heart, liver, and epididymal adipose tissues were quickly dissected and snap frozen in liquid nitrogen. The tissues were homogenized and activation of insulin receptor and Akt by insulin was analyzed by immunoblotting.

### Immunoblotting

Preparations of tissue lysates, protein determination, SDS-PAGE and transfer of SDS gels to Immobilion-FL membranes (Millipore) were performed as previously described (Viswambharan et al., 2007). The resultant membrane was stained with ponceau S and first incubated with the corresponding primary antibody at room temperature for 2 h with gentle agitation after blocking with 5% skimmed milk. The blot was then further incubated with a corresponding anti-mouse (Alexa fluor 680 conjugated) or anti-rabbit (IRDye 800 conjugated) secondary antibody. Signals were visualized using Odyssey Infrared Imaging System (LI-COR Biosciences). The phosphorylated form of insulin receptor-β or Akt was normalized to corresponding ponceau signals. Quantification of the signals was performed using NIH Image 1.62 software.

### *In-situ en face* Nitric oxide production in aortas

Nitric oxide (NO) production in response to insulin in aortas was assessed by *in-situ en face* staining of NO with the membrane-permeable dye DAF-2DA. Briefly, mouse thoracic aortas cleaned of perivascular tissues were equilibrated for 30 min in Krebs buffer supplemented with L-arginine (100 μmol/L) at 37°C and aerated with 95% O_2_ and 5% CO_2_. DAF-2DA (5 μmol/L) was then added to the buffer for 30 min. The aortas were then washed three times with fresh Krebs-Ringer buffer and stimulated with insulin (1 μmol/L) for 20 min. The aortic rings were then immediately fixed in cold 4% PFA solution for 30 min and carefully opened longitudinally, counterstained with DAPI (1 μg/mL, 1 min) and mounted *en face* for endothelial layer imaging. Vectashield mounting medium was used to preserve the fluorescence. The fluorescence was analyzed in a Leica DM6000 confocal microscope within hours after preparation. DAF-2DA was excited with 488 nm argon laser, and images were collected at 500–535 nm emission. At least 4–5 fields were taken from each vessel segment. The images from DAF-2DA and DAPI staining were quantified with Image J software (U. S. National Institutes of Health) and results are presented as the ratio of DAF-2DA and DAPI positive nucleus.

### Hepatic glycogen content

For the liver glycogen determination, small liver pieces were weighed and adjusted to a final concentration of 0.1 mg liver tissue per μl with 2 M hydrochloric acid (Schmutz et al., 2010). Samples were sonicated and incubated for 3 h at 99°C with constant agitation to break down glycogen in glucose and glucose-6-phosphate. After neutralization with an equal volume of 2 mol/L NaOH and 8 volumes of 100 mmol/L Tris-HCl pH 7.4, glucose levels were determined in 20 μl of neutralized solution using the glucose-hexokinase kit (Sigma-Aldrich). To visualize glycogen in liver sections, 7 μm-thick paraffin-embedded liver sections were stained with Periodic Acid-Schiff (PAS) reagent using standard protocols followed by counterstaining with Mayer's haematoxylin solution (Sigma-Aldrich) according to the manufacturer's instruction. A part of the liver tissues from WT and *mPer2*^*Brdm1*^ mice were snap frozen in liquid nitrogen and homogenized for immunoblotting analysis of glycogen synthase expression.

### qRT-PCR of inflammation markers in adipose tissues

RNA was extracted out of high fat tissue by homogenizing the tissue directly in TRI Reagent® RNA Isolation Reagent followed by phase separation with chloroform, RNA precipitation with Isopropanol and RNA washing in 75% Ethanol. First-stranded cDNA was synthesized from 500 ng total RNA by using a random primer and reverse transcriptase. Real-Time PCR was performed with the IQTM SYBR Green Supermix and iCycler system (Bio-Rad). Amplification was achieved using an initial cycle of 95°C for 10 min, followed by 40 cycles of 95°C for 15 s, and 60°C for 1 min. Specificity of PCR was checked by melting curve analysis. Expression of mRNA was normalized to the reference gene glyceraldehyde-3-phosphate dehydrogenase (GAPDH). Real-time primers (f, forward; r, reverse) used to detect expression of the corresponding murine genes were as follows: IL-6 f: GACAACCACGGCCTTCCCTA, r: GCCTCCGACTTGTGAAGTGGT; TNF-α f: GGCAGGTCTACTTTGGAGTCATTGC, r: ACATTCGAGGCTCCAGTGAATTCGG; MCP-1 f: AGCACCAGCCAACTCTCAC, r: TCTG GACCCATTCCTTCTTG; F4/80 f: TGGCTGCCTCCCTGACTTTC, r: CAAGATCCCTGCCC TGCACT; CD11c f: GGAGGAGAACAGAGGTGCTG, r: CACCTGCTCCTGACACTCAA; GAPDH f: ACCCAGAAGACTGTGGATGG, and r: ACACATTGGGGGTAGGAACA.

### Statistical analysis

Results are presented as means ± SEM. In all experiments, *n* equals the number of animals. Statistical analysis was performed with Student's *t*-tests for unpaired observations, or by One-Way ANOVA for multiple comparisons followed by Bonferroni adjustment. A two tailed *p*-value smaller than 0.05 is considered statistically significant.

## Results

### Compromised insulin receptor activation in *mPer2*^*Brdm1*^

**Figure 1:**
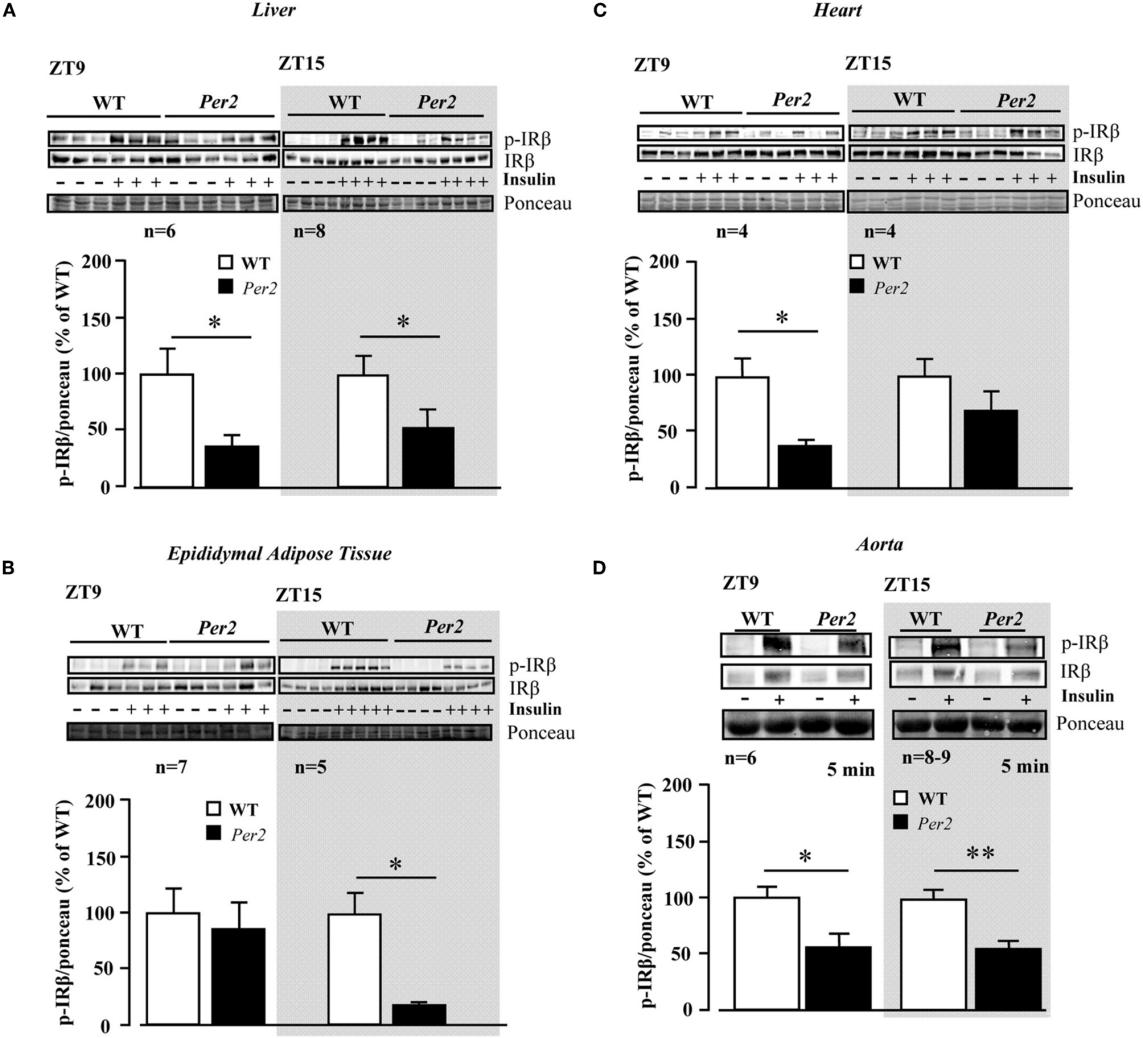
**Compromised insulin receptor activation in response to insulin in the liver (A), epididymal adipose tissue (B), heart (C), and aortas (D) of *mPer2*^*Brdm1*^ mice.** Representative immunoblotting and respective quantifications of insulin receptor β subunit phosphorylations (Y1162/1163) upon intraperitoneal injection of insulin (0.5 mU/g body weight, 5 min) at ZT9 and ZT15, respectively (*n* = 4−8). ^*^p < 0.05 between indicated groups. Aortas are stimulated by insulin (100 nmol/L, 5 min) *ex vivo* at ZT9 and ZT15. The symbol “−” or “+” means without or with insulin stimulation, respectively. ^*^ = *p* < 0.05 and ^**^p < 0.01 between the indicated groups.

Upon intraperitoneal injection of insulin (0.5 mU/g body weight, 5 min), activation of insulin receptor as measured by autophosphorylation of the β receptor subunit at tyrosine (Tyr)-1162/1163 was monitored in various tissues in both WT and *mPer2*^*Brdm1*^ mice (Figure 1). As compared to WT mice, the insulin receptor activation in the liver of *mPer2*^*Brdm1*^ mice was compromised at both ZT9 and ZT15 (Figure 1A). The decreased insulin receptor activation was also observed in the epididymal adipose tissue of the *mPer2*^*Brdm1*^ mice at ZT15 (Figure 1B) and in the heart at ZT9 (Figure 1C). In the aortas, activation of the insulin receptor by *ex vivo* stimulation with insulin at 100 nmol/L for 5 min was significantly reduced in *mPer2*^*Brdm1*^ mice at both ZT9 and ZT15 as compared to WT controls (Figure 1D).

### Compromised insulin stimulated Akt activation in *mPer2*^*Brdm1*^

**Figure 2:**
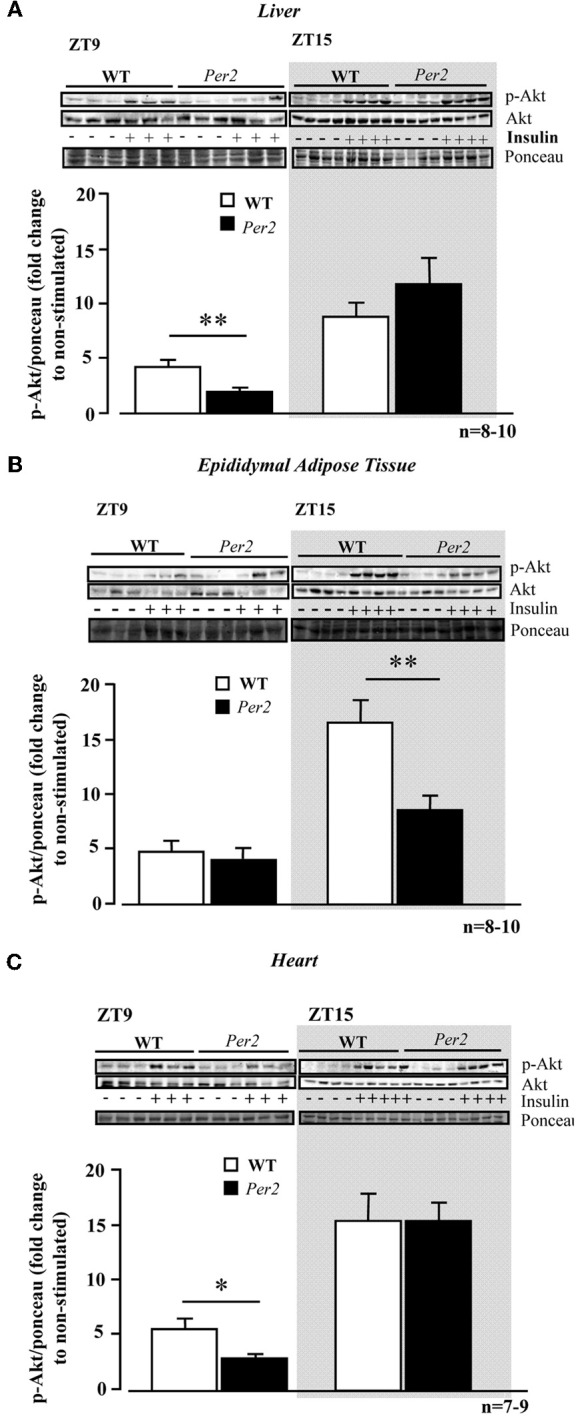
**Compromised Akt activation in response to insulin in the liver (A), epididymal adipose tissue (B), and heart (C) of *mPer2*^*Brdm1*^ mice.** Representative immunoblotting and respective quantifications of Akt-S473 phosphorylation upon intraperitoneal injection of insulin (0.5 mU/g body weight, 5 min) at ZT9 and ZT15 (*n* = 7−10), respectively. The symbol “−” or “+” means without or with insulin stimulation, respectively. ^*^p < 0.05 and ^**^p < 0.01 between the indicated groups.

**Figure 3:**
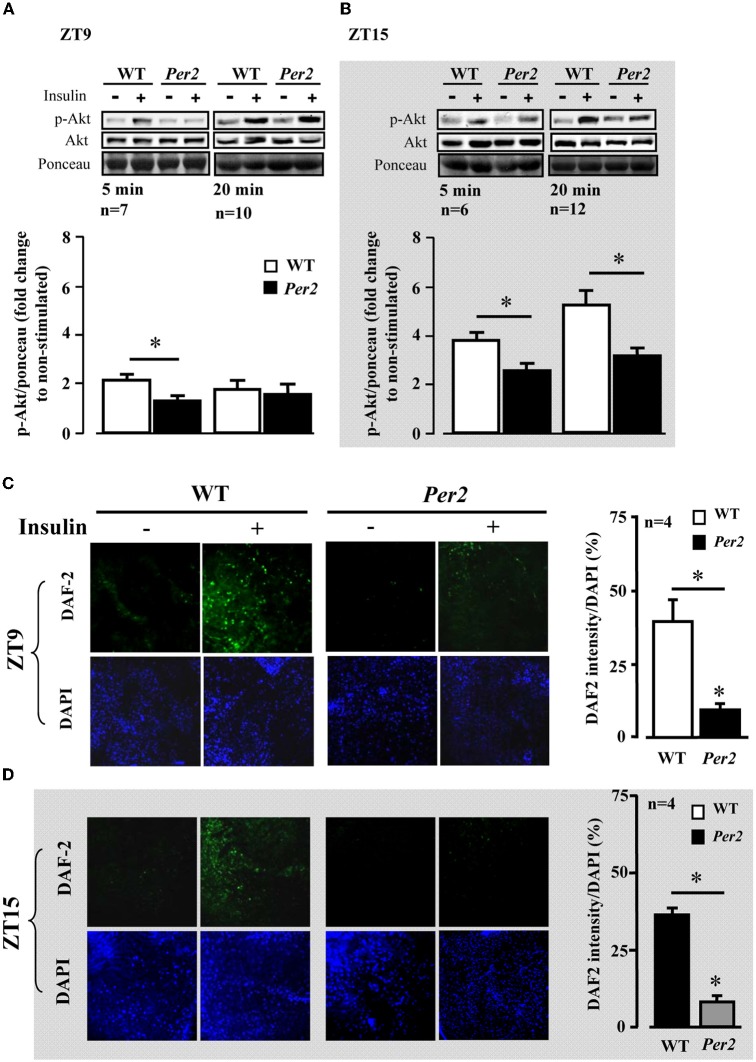
**Impaired aortic Akt activation and NO release in response to insulin in *mPer2*^*Brdm1*^ mice.** (**A** and **B**) Representative immunoblotting and respective quantifications of Akt-S473 phosphorylation stimulated by insulin (100 nmol/L, 5 or 20 min as indicated) at ZT9 and ZT15, respectively, in aortas of WT and *mPer2*^*Brdm1*^ mice. (**C** and **D**) Confocal microscopic analysis of insulin-stimulated NO production in aortic endothelial layer measured by *en face* DAF-2DA staining followed by DAPI nucleus counterstaining at ZT9 and ZT15, respectively. The bar graph in the right panel shows the respective quantification of NO signals. Results are presented as ratio of DAF-2DA fluorescence intensity to DAPI (*n* = 4). ^*^ = *p* < 0.05 between the indicated groups. The symbol “−” or “+” means without or with insulin stimulation, respectively. Bar = 200 μm.

We further analysed Akt activation upon insulin stimulation in the above mentioned tissues. In the liver of *mPer2*^*Brdm1*^ mice, there was a significant decrease in Akt activation as measured by Akt-S473 phosphorylation in response to insulin at ZT9, but not at ZT15 as compared to the WT mice (Figure 2A). In the epididymal adipose tissue, impaired Akt activation upon insulin stimulation was observed at ZT15 in the *mPer2*^*Brdm1*^ mice (Figure 2B) and in the heart at ZT9 (Figure 2C). In the aortas, Akt-Ser-473 phosphorylation level upon insulin stimulation (100 nmol/L, 5 min) at ZT9 was significantly lower in the *mPer2*^*Brdm1*^ as compared to the WT mice, but no significant difference was observed 20 min after insulin stimulation (Figure 3A), indicating a delayed Akt activation in aortas of *mPer2*^*Brdm1*^. At ZT15, the activation of Akt stimulated by insulin (100 nmol/L) was significantly reduced in the *mPer2*^*Brdm1*^ mice at 5 and 20 min (Figure 3B). Accordingly, aortic NO production in response to insulin (100 nmol/L; 20 min) as monitored by *en face* DAF-2DA staining in the intact aortas was blunted in *mPer2*^*Brdm1*^ at both ZT9 and ZT15 time points (Figures 3C and D).

### *mPer2*^*Brdm1*^ mice exhibit alterations in glucose homeostasis

**Figure 4:**
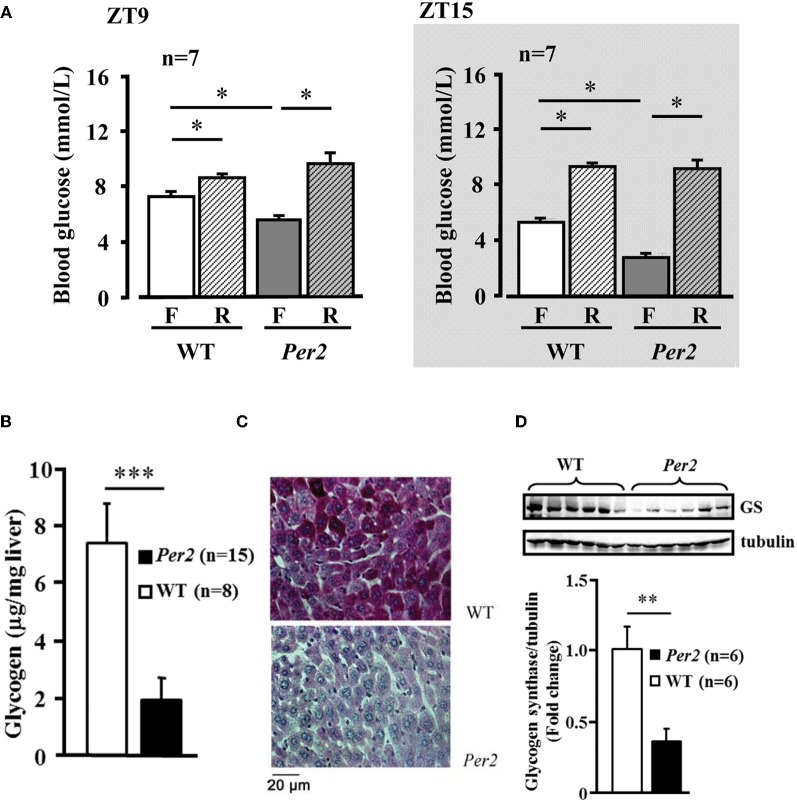
**Decreased fasting plasma glucose concentration and hepatic glycogen content in *mPer2*^*Brdm1*^ mice. (A)** Decreased fasting (F) blood plasma glucose concentration in the *mPer2*^*Brdm1*^ mice as compared to WT littermates at ZT9 and ZT15, which is normalized after refeeding (R). **(B)** Fasting hepatic glycogen content of WT and *mPer2*^*Brdm1*^ mice at ZT9. **(C)** Representative Periodic Acid-Schiff (PAS) staining showing hepatic glycogen contents in WT and *mPer2*^*Brdm1*^ mice. **(D)** Immunoblotting showing fasting hepatic glycogen synthase (GS) levels in WT and *mPer2*^*Brdm1*^ mice. ^*^p < 0.05, ^**^p < 0.01, and ^***^p < 0.005 between the indicated groups, respectively.

**Figure 5:**
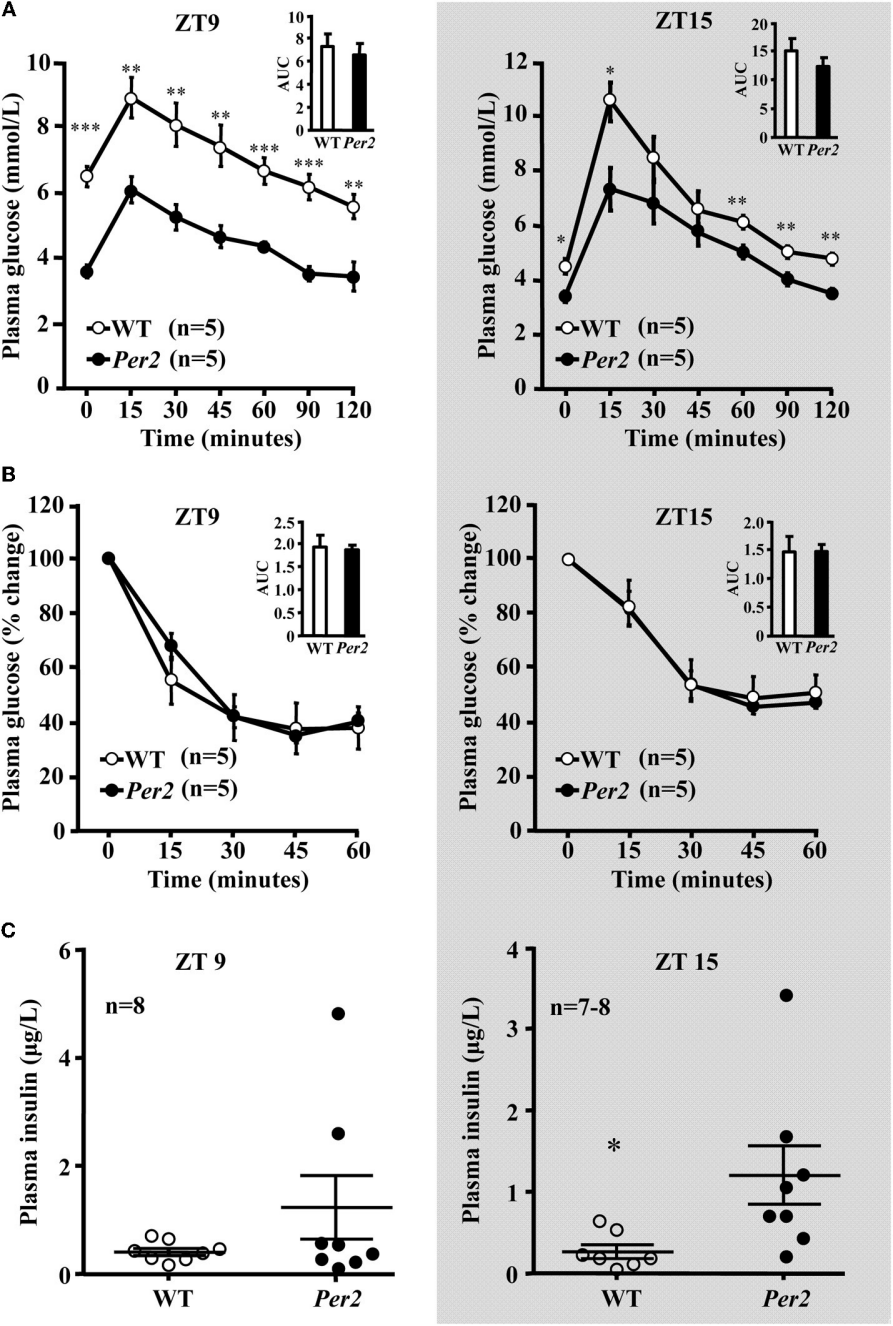
**GTT, ITT test, and basal plasma insulin concentration in *mPer2*^*Brdm1*^ mice. (A)** Glucose tolerance test, **(B)** insulin tolerance test, and **(C)** basal fasting plasma insulin levels in the *mPer2*^*Brdm1*^ and WT mice at ZT9 and ZT15 (one blood sample from a WT mouse was lost during preparation); ^*^p < 0.05 and ^**^p < 0.01, and ^***^p < 0.005 vs. *mPer2*^*Brdm1*^ mice.

In the *mPer*2^*Brdm*^ mice, 6-h fasting plasma glucose concentration was significantly lower compared to the WT animals at both ZT9 and ZT15, which was normalized after refeeding (Figure 4A). Food intake during the refeeding was not different between the two groups (WT: 1.5 ± 0.12, and 1.47 ± 0.17 g/3 h, *n* = 16 in each group). Lower fasting hepatic glycogen content and lower glycogen synthase protein level in *mPer2*^*Brdm1*^ were observed as compared to WT controls (Figures 4B–D). The *mPer*2^*Brdm*^ mice showed no significant difference in glucose tolerance (area under the curve) and insulin tolerance at both ZT9 and ZT15 (Figures 5A and B). The fasting plasma insulin level at ZT15 was significantly higher in the *mPer*2^*Brdm*^ mice as compared to the WT littermates (Figure 5C). The same trend was also observed at ZT9, although it did not reach statistical significance.

### *mPer2*^*Brdm1*^ mice show no difference in HFD-induced obesity

**Figure 6:**
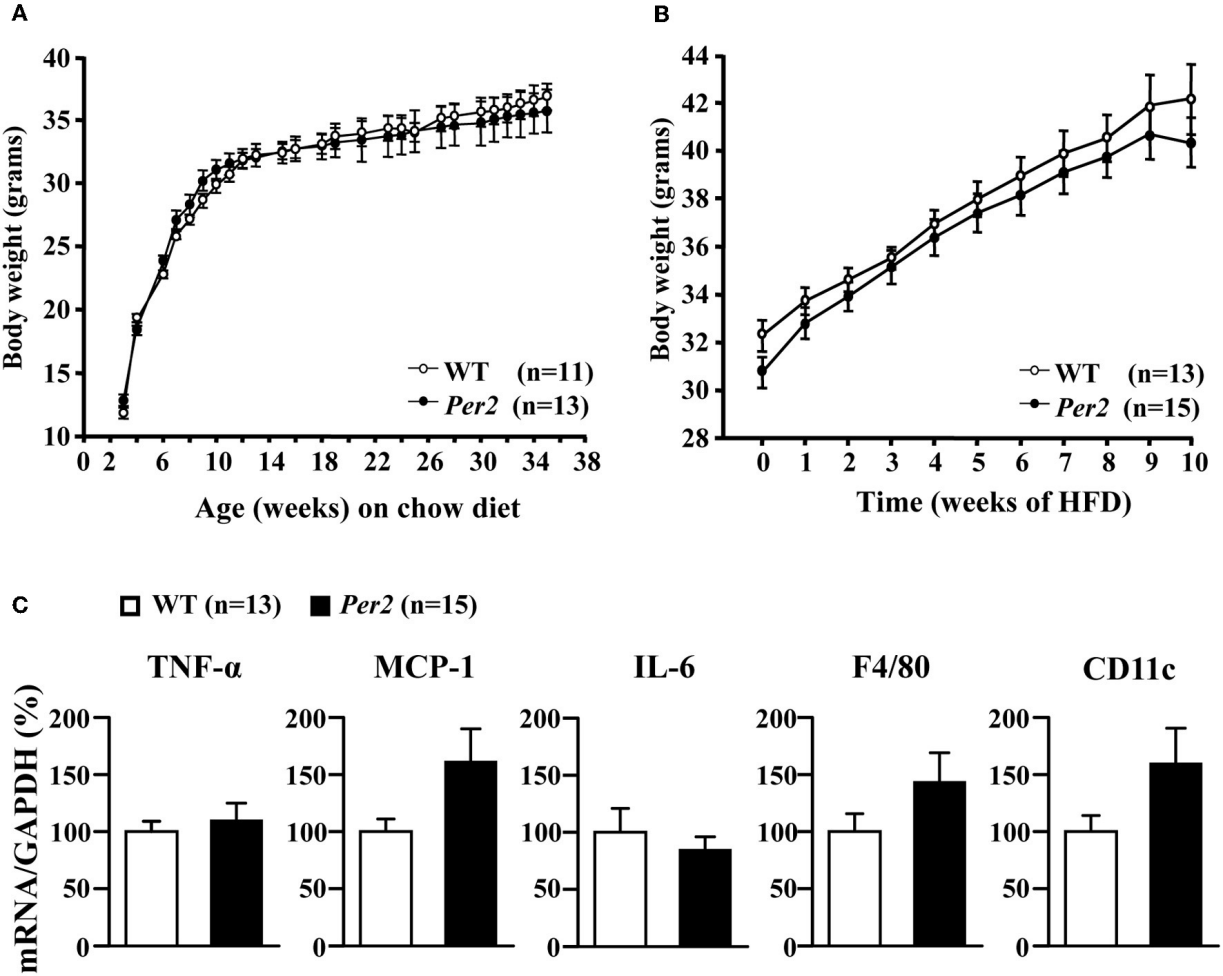
**Body weight development and diet-induced obesity of wild type (WT) and *mPer2*^*Brdm1*^ mice. (A)** Comparable body weight development on chow diet and **(B)** obesity on high fat diet (HFD) feeding between WT and *mPer2*^*Brdm1*^ mice, and (**C**) qRT-PCR shows no significant difference in adipose tissue inflammation markers.

WT and *mPer2*^*Brdm1*^ mice showed similar body weight development on normal chow diet over 36 weeks (Figure 6A) and obesity on high fat diet feeding (starting at the age of 12 weeks) over 10 weeks (Figure 6B). qRT-PCR analysis of epididymal adipose tissues obtained from high fat diet fed mice revealed a trend of increase in several cytokine gene expression i.e., TNF-α, MCP-1, IL-6, and in the macrophage markers F4/80 and CD11c in the *mPer2*^*Brdm1*^ mice as compared to the WT controls (Figure 6C).

## Discussion

In the present study, we show a blunted activation of the insulin receptor upon insulin challenge in various tissues including liver, fat, heart, and aortas of the *mPer2*^*Brdm1*^ mutant mice. The activation of the insulin receptor downstream signaling is affected as evidenced by reduced or delayed Akt phosphorylation. In accordance with our results, a decreased activation of Akt by vascular endothelial growth factor in the endothelial cells has also been reported in the *mPer2*^*Brdm1*^ mice (Wang et al., 2008). This has been thought to be due to increased activity of phosphatases that dephosphorylate Akt in these mice. Similar to *mPer2*^*Brdm1*^ mice, decreased Akt signaling has been observed in mice with functional disruption of other circadian clock genes such as *CLOCK* and *BMAL1* (Anea et al., 2009). The results suggest that insulin signaling and Akt activity are under the control of the circadian clock. An interesting topic which remains to be investigated is the question whether phosphatases are involved in compromised insulin receptor activation in the *mPer2*^*Brdm1*^ mice.

In the vasculature, insulin stimulates NO release through Akt (Montagnani et al., 2001), and decreased insulin-stimulated NO release is associated with obesity (Yu et al., 2011). This represents one of the mechanisms linking obesity to cardiovascular diseases (Bornfeldt and Tabas, 2011). Our previous study demonstrated that mice with a mutation in the *Per2* gene exhibit impaired endothelium-dependent relaxations in response to acetylcholine and Ca^2+^-ionophores (Viswambharan et al., 2007), the agonists that activate eNOS by Ca^2+^/calmodulin-dependent mechanisms. Our present study extends these observations and we show that the *mPer2*^*Brdm1*^ mice exhibit compromised vascular insulin signaling linked to decreased NO production, a process that is independent on the increase in intracellular Ca^2+^ concentration mediated by Akt (Montagnani et al., 2001). The impaired activation of Akt in the aortas upon insulin stimulation reveals a specific diurnal pattern. It is significantly decreased in the aortas of *mPer2*^*Brdm1*^ mice at both ZT9 and ZT15, although the pattern of reduced Akt activation is slightly different. The endothelial dysfunction in *mPer2*^*Brdm1*^ mice has been shown to be associated with accelerated cellular senescence, impaired angiogenic capacity, and increased susceptibility to ischemia-induced injury in the hind limbs (Wang et al., 2008). The results from our previous study and the present study suggest that besides an intrinsic defect of eNOS enzymatic activation, in the *mPer2*^*Brdm1*^ mice, the insulin signaling leading to eNOS activation is compromised as well. Further experiments will focus on the elucidation of the underlying molecular mechanisms of insulin signaling regulated by the *Per2* gene. The effect of circadian gene mutation on vascular endothelial function may explain circadian variation of the onset of cardiovascular events such as myocardial infarction, sudden cardiac death, and stroke in human populations (Muller et al., 1985; Cohen et al., 1997).

The heart must adapt rapidly to changes in circulating energy supply. It has been suggested that the circadian clock in the heart is essential for cardiac gene expression, responsiveness of the heart to energy substrates, and contractile function (Young, 2006). A recent study demonstrates that cardiac function in mice with disruptions in circadian clock genes is significantly attenuated under an imposed light regimen (Wu et al., 2011). The effects of the *Per2* gene on heart function are not clear. Our previous studies showed no effects of the *Per2* mutation on ECG in mice (Viswambharan et al., 2007). The *mPer2*^*Brdm1*^ mice display, however, a mild cardiovascular phenotype *in vitro* despite endothelial dysfunction with an elevated 24-h heart rate, a decreased 24-h diastolic blood pressure, and an attenuation of the dark–light difference in blood pressure and heart rate (Vukolic et al., 2010). In the present study, we show compromised insulin receptor activation and Akt activation in the heart of the *mPer2*^*Brdm1*^ mice. The role of insulin in the heart is to regulate cardiomyocyte energy metabolism, including fatty acid and glucose uptake, increase cardiac efficiency (cardiac performance/oxygen consumption) and protein synthesis, stimulate angiogenesis, suppress apoptosis, to promote cell survival, and increase blood perfusion of myocardium, principally through Akt (Iliadis et al., 2011). The impact of the compromised insulin signaling in the heart of the *mPer2*^*Brdm1*^ mice observed in our present study under physiological and pathological conditions warrants further investigation.

Another interesting phenotype of the *mPer2*^*Brdm1*^ mice reported in this study is lower fasting hepatic glycogen content and glycogen synthase expression, suggesting the involvement of *Per2* gene in regulation of fasting liver glycogen metabolism. The liver is mostly responsible for maintaining plasma glucose levels during fasting. Under the fasting condition, the liver produces more glucose via glycogenolysis and gluconeogenesis, which is regulated by insulin (Peeters and Baes, 2010). The lower fasting liver glycogen content and lower glycogen synthase levels explain the lower fasting plasma glucose concentration in the *mPer2*^*Brdm1*^ mice and suggest an impaired ability of the liver to store glucose in the form of glycogen. Further research should address the question whether this is linked to compromised insulin receptor activation and Akt activation in the liver of the *mPer2*^*Brdm1*^ mice, and how *Per2* gene regulates liver glycogen metabolism. It is to note that blood glucose levels in the *mPer2*^*Brdm1*^ mice after refeeding are comparable to those in the WT mice, but remain low after a defined glucose load (GTT). An enhanced food intake in the mutant mice can be ruled out, since food intake during refeeding is not different between the two groups. It is to speculate that gastrointestinal hormonal regulation mechanisms of glucose homeostasis (Maggs et al., 2008) might be altered in the *mPer2*^*Brdm1*^ mice, which requires thorough investigation.

Insulin receptor-mediated Akt signaling in insulin sensitive tissues is crucial for glucose homeostasis (Bornfeldt and Tabas, 2011). A decrease in insulin receptor signaling mediates reduced responsiveness of tissues or cells to insulin, i.e., insulin resistance, which is central for development of metabolic syndrome and type-II diabetes. It is accompanied by chronic low grade inflammation manifested by inflammatory macrophage infiltration and pro-inflammatory cytokine production in adipose tissues (Weisberg et al., 2003; Xu et al., 2003; Lumeng and Saltiel, 2011). To our surprise, the lower fasting plasma glucose level in the *mPer2*^*Brdm1*^ mice is accompanied with higher plasma insulin levels and comparable insulin tolerance test and glucose tolerance test compared to WT control mice. These results suggest that the lower fasting plasma glucose level in the *mPer2*^*Brdm1*^ mice may result from higher pancreatic insulin secretion that compensates the reduced insulin signaling in various peripheral insulin sensitive tissues in the *Per2* mutant mice.

It is to be noted that there is a difference in the activation of the insulin receptor or Akt among different tissues at identical ZT time point. For example, insulin receptor activation in the *mPer2*^*Brdm1*^ mice is impaired at ZT9 in the liver, heart, aortas, but not in the epididymal adipose tissue. This may be due to a tissue specific insulin response profile or to tissue specific regulatory effects of the *Per2* gene on insulin responses. Furthermore, there is a discrepancy between insulin receptor activation and Akt activation in the same tissue, e.g., activation of the insulin receptor in the liver of *mPer2*^*Brdm1*^ mice is reduced at both ZT9 and ZT15, while Akt activation is reduced only at ZT9. The underlying mechanism is not known. Whether this is due to specific phosphatase activities that regulate insulin receptor activation and Akt activation and whether it is differently affected by lack of the *Per2* gene remains to be investigated. Finally, there is also a differential activation of insulin receptor and Akt between ZT9 and ZT15. This could be explained by the possibility that PER2 regulates the insulin signaling pathway in a time dependent manner.

Studies from the literature with *Per2* gene modified mice reported conflicting results regarding predisposition of the mice to obesity. A study using *mPer2*^*Brdm1*^ mice showed that they are more susceptible to obesity when they are fed HFD (Yang et al., 2009), while another study reported decreased body weight development and adiposity (Grimaldi et al., 2010). It is even more puzzling that the *Per*2^−/−^ mouse embryonic fibroblasts (MEF) showed increased adipocyte differentiation (Grimaldi et al., 2010). In contrast to these studies, our present report demonstrates that upon HFD feeding for 10 weeks, the *mPer2*^*Brdm1*^ mice do not reveal any difference in body weight gain as compared to WT control mice, suggesting that the *Per2* gene mutation does not have an impact on growth development and predisposition to obesity. Additionally, no significant difference in obesity-associated adipose tissue inflammation markers has been observed between obese WT and *mPer2*^*Brdm1*^ mice on HFD, although there is a trend of increased inflammation markers in the obese *mPer2*^*Brdm1*^ mice. These findings are in line with our previous observation that the *mPer2*^*Brdm1*^ mice exhibit no difference in food intake under normal chow or HFD *ad libitum* feeding and no change in body compositions, although these mice did not show food anticipatory activity under the condition of caloric restriction (Feillet et al., 2006). The discrepancy between our findings and others on obesity predisposition of *Per2* gene disrupted mice is not clear. It is of note that only results on female mice were reported (Yang et al., 2009) and our study was performed in male mice. Moreover, whether the different phenotypes of mice are due to *Per2* gene allele ablation or mutation requires further investigation.

In conclusion, our study shows that mice with a mutation in the circadian clock gene *Per2* have compromised insulin signaling, associated with lower hepatic glycogen content and decreased vascular endothelial function, but no increased predisposition to obesity. These results suggest a critical role of the *Per2* gene in the regulation of glucose homeostasis particularly under food restricted conditions or in diabetes, since patients with diabetes exhibit decreased rates of glycogen synthesis and decreased glycogen contents in the liver (Krssak et al., 2004). Our study suggests that *Per2* gene could play a role in regulation of glucose homeostasis in diabetes particularly through the regulation of hepatic glycogen contents.

### Conflict of interest statement

The authors declare that the research was conducted in the absence of any commercial or financial relationships that could be construed as a potential conflict of interest.
